# The Automatic Methods Group *Newsletter*

**DOI:** 10.1155/S1463924696000065

**Published:** 1996


Journal of Automatic Chemistry, Vol. 18, No. 2 (March–April 1996), pp. 59–69

AUTOMATIC METHODS GROUP

Analytical Division
ROYAL SOCIETY OF CHEMISTRY

### The Automatic Methods Group *Newsletter*

The AMG Newsletter is published as part of and in collaboration with the *Journal of Automatic Chemistry*. Further information on the *Journal of Automatic Chemistry* can be obtained from Taylor & Francis Ltd, Rankine Road, Basingstoke, Hampshire RG24 8PR or e-mail: info@tandf.co.uk; worldwide web home page: http: ||tandf.co.uk.

### Monitoring the needs of society: New horizons in pollution control

*A joint meeting of the Automatic Methods Group and South East Region of the Royal Society of Chemistry in co-operation with the Health and Safety Executive was held on 12 and 13 December 1995 at the Scientific Societies Lecture Theatre, New Burlington Place, London W1. Abstracts of some of the papers read at the meeting and the posters presented follow. The meeting was divided into four sessions:*

*Session 1: Directives and standards*

*Chairman: R. H. Brown*

*Session 1(a) Workplace monitoring*

*Basis for the legislation and guidance*
*Mike Mahony*

*Practical applications*
*Jeff Friar*

*Moves towards standardization in the USA*
*Martin Harper*

*Session 1(b) Indoor air*

*Indoor air: basis for legislation and guidance*
*Derek Crump*

*Analysis of VOCs in indoor air: practical applications*
*Jan Kristensson*

*Session 1(c) Environmental monitoring*

*Chairman: J. R. P. Clarke*

*The basis for the legislation and guidance*
*Stephanie Coster*

*The use of diffusive sampling for monitoring the environment*
*Kevin J. Saunders*

*Session 2: Framework for measurement*

*Quality and accreditation*
*Norman West*

*Calibration and traceability*
*Theo L. Hafkenscheid*

0142-0453/96 $12.00 © 1996 Taylor & Francis Ltd.

*Session 3: Measurement*

*Analysis of organic volatiles by thermal desorption*

*Introduction to thermal desorption techniques*
*Richard H. Brown*

*Developments in multi-bed sorbents*
*Greg Johnson*

*Chairman: K. J. Saunders*

*Using hand-held electronic instruments*

*An introduction*
*Peter T. Walsh*

*New advances in sensor technology*
*David Williams*

*Use of detector tubes in the monitoring of workplace atmospheres*
*Stefan Zloczysti*

*Recent advances in specific benzene detection with colorimetric spot measurement systems*
*R. S. Pulz and W. May*

*Strategy for urban monitoring*
*Frank R. Price*

*The United Kingdom air quality monitoring networks*
*Clare Downing*

*Session 4: New horizons for pollution control*

*Chairman: Alan Braithwaite*

*A new piezo-electric ozone monitor*
*Emile de Saeger and P. Pérez Ballesta*

*Portable XRF systems for pollution monitoring*
*Margaret West*

*The electret dust sampler*
*Richard C. Brown*

*Portable monitors for asbestos*
*Garry Burdett*

### Basis for the legislation and guidance

**Mike Mahony**
*Health and Safety Executive, London*

There are three main relevant pieces of health and safety legislation in the UK—COSHH, CAW and CLAW. The latter two apply specifically to asbestos and lead but COSHH applies generally to all other types of substances hazardous to health, including biological agents. COSHH is likely to be amended in the future to implement the Amendment to the Carcinogens Directive, and possibly—subject to negotiations in the EC—the proposed Chemical Agents Directive.

COSHH, introduced in 1988, was regarded as a pioneering piece of occupational health and safety legislation. It was the first goal-setting set of regulations dealing with the general problem of substance-induced ill-health in the workplace, being based on risk assessment leading to appropriate measures to prevent or control exposure to hazardous substances. It replaced a large number of prescriptive and industry-specific regulations.

The principles underlying COSHH are risk assessment; planning (of risk reduction and control measures); implementing; monitoring; modifying, on a par with the planning cycle familiar from management theory. COSHH also introduced a hierarchy of control measures: exposure to hazardous substances must be prevented, or if prevention is not reasonably practicable, controlled by means other than personal protective equipment (PPE); but PPE must also be used if control by other means alone is not reasonably practicable.

Under COSHH, control of exposure has to be adequate, and adequate control is defined by COSHH as including compliance with occupational exposure limits for substances which present a risk to health by inhalation. There are two types of limits: occupational exposure standards (OESs) and maximum exposure limits (MELs). These relate to the time-weighted average concentration in air of the substance to which employees are exposed over a period. An OES is a level scientifically recognized as safe. An MEL is set for a substance which has no such recognized safe level, or for which a recognized safe level is not reasonably practicable to attain. OESs may be temporarily exceeded subject to certain conditions, but MELs may not and exposure must be reduced as far below a MEL as reasonably practicable.

The list of MELs and OESs is published annually in a guidance document known as EH 40. Other guidance on COSHH includes nine Approved Codes of Practice (ACoPs), but the main ones which need concern us are the General ACoP and the Carcinogens ACoP. These give statutory guidance on meeting the goal-setting requirements of the COSHH Regulations. Other guidance on COSHH covers COSHH assessments, and substitution. Sector specific guidance on COSHH is also available for particular industries.

CAW and CLAW similarly include requirements to keep the concentration in air of asbestos fibres or lead within limits, and are likewise supported by ACoPs and guidance.

### Practical applications

**Jeff Friar**
*Health and Safety Executive, Bootle*

Substances in the workplace may enter the body by inhalation, by passing through the intact skin or by ingestion. The Control of Substances Hazardous to Health (COSHH) and similar legislation is rooted in the need to provide individuals in the workplace with adequate control of exposure. Whether control is adequate or not requires a judgement on whether such control is commensurate with the risks. Thus there is a need to assess risk, to decide on what controls are necessary to address this risk, to provide these controls and, finally, to ensure that the controls really are adequate and that they continue to be so. All of this may require measurement.

'Hazard' is the potential to cause harm and 'risk' is the chance of that harm occurring. For substances hazardous to health, risk is dependent upon exposure. Measurement is most often associated with exposure by inhalation, which is defined as the concentration of substance in the breathing zone over either 8 hours or 15 minutes. Exposures defined in this way take no account of the use of respiratory protective equipment (RPE). In risk assessment, exposure may be compared to a level of concern which may be expressed as an Occupational Exposure Limit (OEL).

It is not enough simply to measure—exposure is often very complex. Measured exposure data should properly represent exposure over the sampling period and should be capable of properly representing exposure throughout the whole of the time weighted average reference period. There should be sufficient information to place exposures in context. The frequency and duration of exposure may also be important. The data should be collected following good occupational hygiene practice, preferably employing standardized procedures. Meaningful measurements of exposure can only be made by paying close attention to such practical factors.

### Moves towards standardization in the USA

**Martin Harper**

*SKC Inc., USA*

As the end of the century approaches, many long-held paradigms are being evaluated, and opened to change. In the USA, one such paradigm under close scrutiny is the role of government in regulation. This a particularly true in the field of occupational and environmental hygiene, and in the narrower field of air sampling instrumentation, and, in part, re-evaluation is related to the view that government should not interfere in the market forces that cause industries to be self-regulating. In the early phase of air sampling instrumentation development, performance standards were laid down by NIOSH (the NIOSH Accuracy Criterion), and adopted by OSHA. NIOSH also published guidelines for performance evaluation (the 'blue pages'). Unfortunately, this situation has been complicated by the introduction of separate standards from OSHA, MSHA, and the EPA, and the resulting confused situation has been severely tested by the introduction of the Comité Européen de Normalisation (CEN) performance standards in Europe. Government agencies in the US have accepted the position that existing standards require updating and consolidation. Much of this work is being undertaken by private bodies such as ASTM and ANSI, where both government and manufacturers may find a common forum. Government has the advantage of being able to reference independent standards, without going through the tortuous procedure of promulgating their own. Manufacturers find that, by being included in the rule-making process, they are able to ameliorate the costs and burdens of meeting the standards. This appears to be similar in concept to the Working Groups and Technical Committees of CEN, with the drawback that extra funding to involve experts without a financial stake is rarely available. Therefore, an important part of all standards is considered to be the paper review process. Since the CEN has given the US a lead, much of their experience can be incorporated, and, with their continued assistance and goodwill, the US can strive for standards that might ultimately be acceptable on both sides of the Atlantic, for incorporation as ISO standards.

### Indoor air: basis for legislation and guidance

**Derrick Crump**
*Building Research Establishment, Garston*

Indoor air pollution is the presence within buildings of toxic or other substances which may, directly or indirectly, be a cause of ill health or discomfort. In developed countries with cold and/or temperate climates, people on average spend 90% of their time indoors and 75% of that in the home. Vulnerable groups such as the sick, the very young and elderly may spend over 90% of their time in the home. It follows therefore that exposure to indoor air pollutants can form a significant proportion of the total exposure of an individual to these substances.

Good air quality relies to a certain extent on clean air being available from outside the building. Within the building itself and, more exceptionally, in the ground are many sources of chemicals that may add to the background level due to the outside air. These are: emissions from building materials, furnishings and fittings; pollutants from the ground; moisture that can encourage degradation of materials and mould; activities of people resulting in the release of chemicals including the use of consumer products and smoking of tobacco; combustion products from heating and cooking appliances; metabolic products from the occupants themselves.

Measures to control and reduce indoor pollution include housing and public health legislation, building regulations, restrictions on the manufacture, import or use of certain substances, British or international standards, codes of practice and approvals schemes and information and advice to industry and the public. The controls available are focused on new construction and product standards. Within the UK's Building Regulations there are requirements for precautions to be taken to guard against hazardous substances from the ground; the ingress of toxic fumes, particularly formaldehyde; requirements for ventilation to restrict the accumulation of moisture and pollutants; and adequate discharge of combustion products from heating appliances.

It is likely that the Construction Products Directive (Directive 89/106/EEC) will have an increasing impact on the control of sources of indoor air pollutants. Building work must satisfy a number of essential requirements including that of hygiene, health and environment which requires the provision of good indoor air quality. Standards are being produced within the European standardization committees to assess chemical emissions from construction products. Environmental labelling schemes are also likely to bring related benefits of lower emissions of chemicals into the indoor environment.

### Analysis of VOCs in indoor air: practical applications

**Jan Kristensson**
*Chemik Lab AB, Sweden*

'Sick Buildings' are buildings where the occupants complain of discomfort, and Sick Building Syndrome (SBS) is now an accepted problem. The cause of SBS has been attributed to a number of different factors, one of which is poor indoor air quality.

Indoor air quality is dependent on many factors, such as low ventilation efficiency, inadequate building materials, damp accumulation causing chemical reactions in materials or microbial activities, and the activities of the occupants. SBS is probably caused by a combination of several of these factors.

Other problems have also been reported from Sick Buildings, for example increased allergic reactions. Most of the physical factors known to cause SBS, also produce poor indoor air quality and result in an increased concentration of volatile organic compounds (VOCs) in the indoor air. This increase is also responsible for the smell commonly experienced in Sick Buildings. The concentrations of different individual components are usually low, well below the occupational limit values, and usually no toxicological or pathological effects can be measured on the occupants of Sick Buildings. However, technical measurements of VOC concentration, concentrations of indicator compounds, $CO_2$, ventilation efficiency, temperature and humidity can be taken and results from these correlated statistically to results from questionnaires. In this way, practical limits for different compounds and parameters can be statistically established.

It is very important to understand that some of these statistically established practical limits are not general but are correlated to a specific measuring technique. Results obtained from practical applications and the interpretation of these results were discussed in this presentation.

### The basis for the legislation and guidance

**Stephanie Coster**
*Department of the Environment, London*

The Environment Act received Royal Assent on 19 July 1995. Part IV of the Act and Schedule 11 contain provisions for the assessment and management of air quality. Under the Provisions of Section 80, the Secretary of State will, after wide consultation, publish a national air quality strategy. The strategy will include the Government's policies on air quality and will also be a means of implementing air quality provisions of EC Directives and international agreements. It will set out general standards and objectives on air quality and measures which the Government will take and which it looks to local authorities and others to achieve.

Under Section 82, local authorities, as defined in Section 91 (primarily district councils), will be required to undertake periodic reviews within their area of the quality of the air. The review will need to assess the present quality of the air and the likely future quality. For example, if the quality is likely to fall below prescribed standards, the local authority will need to identify the areas where that will apply.

If reviews show that air quality standards or objectives are not being met or are unlikely to be met within a target period, specified in regulation or the national strategy, the local authority will be required, under Section 83, to designate by order the area or areas concerned as an 'air quality management area'.

Local authorities will then have to prepare a written action plan of measures it will take, in full consultation and within the powers exercisable by the authority, and a timetable for implementation to ensure the achievement of the standards and objectives within the designated air quality management area.

### The use of diffusive sampling for monitoring the environment

**Kevin J. Saunders**
*KERIS Ltd, Hook*

Diffusive samplers have been used for workplace monitoring for many years. The first recorded device was the open tube monitor described by Palmes *et al.* in 1973 for sulphur dioxide. Many other devices followed and the technique is now accepted as a fundamental procedure for occupational hygiene measurements.

However, during the last 10 years there has been an increasing need to monitor the environment for airborne contaminants to ensure compliance with EC guidelines and those set by local regulatory authorities.

Diffusive monitors are ideal for this application because of the advantages they offer. They do not require a pump for their operation. They are simple and easy to use, need a minimum of service and have no power requirements. Diffusive monitors can provide a time integrated sample over periods as long as 28 days and can have a limit of detection as low as less than 1 ppb v/v.

In this presentation, the role of diffusive samplers in the monitoring of ambient atmospheres was discussed. Examples and results from large scale surveys for both inorganic and organic pollutants were presented. These include results from long-term surveys over periods of up to four years for the species sulphur dioxide, nitrogen dioxide and C3–C10 hydrocarbons including benzene.

### Quality and accreditation

**Norman West**
*Health and Safety Executive, Sheffield*

Why is there such emphasis today on quality and accreditation in analytical measurement? Essentially, it is because important decisions are made on the basis of analytical results. At best an inaccurate result is a waste of time and money; at worst it could lead to loss of business if a product fails to meet specification, loss of freedom if a forensic analysis is wrong, or even loss of life when dealing with toxic substances. However, it is important to recognize that there is no absolute measure of quality in analytical terms; rather, it depends on the end use made of the results. There are very different requirements for, say, a special steel analysis, as opposed to a trace contaminant in the environment.

Regardless of the requirements, the same well-established principles apply to ensure results that are fit for purpose:

(1) Validated methods, properly documented.
(2) Qualified and trained staff.
(3) Quality system incorporating internal quality control and external quality assurance.
(4) Independent assessment of the quality system.

The application of these principals in the field of occupational hygiene was discussed with particular reference to the WASP proficiency testing scheme and the role of NAMAS accreditation.

### Calibration and traceability

**Th. L. Hafkenscheid**
*Nederlands Meetinstituut, Netherlands*

In general, results of concentration measurements of compounds in the air are used to: pass judgement on current, prevailing situations with respect to air quality or human exposure; extend this judgement to other, current or future situations; and establish relationships with results of other measurement methods or model calculations (validation), and with environmental or health effects (for example through dose-effect relationships or epidemiology).

In order to be able to pass a reliable judgement, or establish a meaningful relationship, the following characteristics must be known:

(1) The representativity of a result in view of its use and the situation being monitored.
(2) The accuracy of the result (how far does the result deviate from the real, 'true' concentration value).
(3) Comparability of the result with results of similar measurements through traceability to one or more generally accepted metrological standards.

This presentation focused on the accuracy and comparability of a measurement result. In practice, it has been found that calibration is a key factor in a measurement that largely determines both accuracy and comparability. Examples of the effects of (lack of) calibration were given and methods for achieving traceability of measurement results were discussed.

### Introduction to thermal desorption techniques

**Richard H. Brown**
*Health and Safety Executive, Sheffield*

An increasingly popular method for the determination of organic volatiles (VOCs) in air is thermal desorption linked to gas chromatography. The method was originally developed for the determination of low-level VOCs in ambient air and for the last 10 years or so has been used extensively for the determination of VOCs in workplace air. More recently, interest has re-emerged in applying the technique to ambient air, as well as for indoor air, and a variety of other areas such as waste tips and the residual solvents in commerical products. Sampling may be either 'actively' by use of a sampling pump or 'passively' by the use of diffusion.

The main advantage of the method over its rival, solvent desorption, is sensitivity, but it also offers improved performance in desorption efficiency, recovery after storage, and ease of automation. However, there are also disadvantages: there is no universal sorbent, and for the more volatile VOCs, the use of a weak sorbent can give rise to back-diffusion when the diffusion sampling mode is chosen.

It is important in the application of thermal desorption techniques to air monitoring that the properties of the sorbents are taken into account. In the case of pumped sampling, the most important parameter is the safe sampling volume, i.e. the maximum air volume that can be sampled before breakthrough of the collected vapour occurs. This volume varies both with the sorbent used and the VOC to be sampled. The presentation discussed the theoretical basis for the calculation and practical measurement of breakthrough volumes, the effect of sampling flow rate and of air humidity. Suitable sorbents were recommended for particular applications and short cuts for predicting breakthrough volumes were discussed.

In the case of diffusive sampling, the most important parameter is the sampling rate, which also varies both with the sorbent used and the VOC to be sampled, although if the sorbent is effectively a 'zero sink', the sampling rate is independent of the sorbent. The presentation discussed the theoretical basis for the calculation and practical measurement of diffusive sampling rates, and the effects of environmental parameters. Suitable sorbents were recommended for particular applications and short cuts for predicting diffusive sampling rates were discussed.

The presentation closed with some discussion on calibration, assessing the validity of thermal desorption methods, and the availability of standard procedures for VOC monitoring.

### Using hand-held electronic instruments—an introduction

**Peter T. Walsh**
*Health and Safety Executive, Sheffield*

There are a wide range of techniques which can be employed to measure the concentration of pollutants in the air. This paper was concerned with the use of hand-held and personal (i.e. unobtrusive monitors which can be attached to personnel) electronic instruments for measurement of pollutants in the workplace and the ambient environment. The principal advantages of hand-held and personal electronic instruments are:

(1) The ability to provide a comprehensive profile of personal exposure by measurement of short-term variations in concentration (assuming an adequate response time for the instrument), peak exposure concentration and duration; and time-weighted-average exposure.
(2) Timeliness by immediate readout of concentration.
(3) Portability and flexibility when undertaking surveys.

Hand-held and personal electronic instruments can be used in a variety of measurement tasks, for example: screening measurements of variation in concentration over time and space; walk-through surveys of workplace, indoor and external environments; measurements near emission sources to determine location and intensity of the source; measurements in confined spaces; concurrent gas monitoring and video filming of worker activity; measuring short-term variations in concentration and peak exposures; investigating the relationship between short and long term exposure; and collecting exposure data for validation of exposure models.

The advantages and limitations of current hand-held and personal electronic instrumentation (for example photo-ionization detectors, flame ionization detectors, semi-conductor sensors, electrochemical cells) for the above tasks were discussed and future trends in gas sensor technology were highlighted.

### Use of detector tubes in the monitoring of workplace atmospheres

**Stefan Zloczysti**

*Auergesellschaft, Germany*

Detector tube systems are widely used in the industry for a variety of applications like screening measurements at the workplace, leak detection in laboratories and factories, and for environmental measurements. However, results obtained by detector tubes are often assessed as being less accurate or less reliable since they may be biased by interferents. New European Standards prepared by CEN/TC 137 in the area of monitoring workplace atmospheres now allow a better selection of procedures and instruments for such measurements. EN 689 (Monitoring Strategy) and EN 482 (Performance Requirements of Methods) specify five major measurement tasks with different requirements for measuring range and overall uncertainty.

An example was given for exposure measurements of solvent concentrations from painting varnish using a combined procedure consisting of a portable photo-ionization detector (PID), pumped sampling tubes and detector tubes. The main paint solvents used for this investigation were xylene, butyl acetate and ethylbenzene. The results produced by a series of measurements in a laboratory test chamber showed a good correlation of all three instruments or methods.

As a result of these tests it was found that subsequent periodic measurements to control exposure of solvents from such paints can be reliably done by means of detector tubes. A specific detector tube calibrated for toluene is suitable when applying an index formula similar to the reciprocal calculation procedure (RCP) as laid down by HSE for mixed solvents.

### Recent advances in specific benzene detection with colorimetric spot measurement systems

**R. S. Pulz and W. May**
*Dragerwerk AG, Germany*

Benzene is becoming increasingly important for both workplace atmosphere and indoor air monitoring with evidence from animal experiments of its potential as a carcinogen. Whereas exposure limit values vary largely between applications and countries, a general trend to lowering thresholds below the 5–10 ppm range can be recognized worldwide. Along with this, the sensitivity of measuring systems must continuously increase to meet the requirements of the respective international norms. Another major challenge results from the need to comply both with a high sensitivity and a high specificity for benzene at high background levels of other hydrocarbons, especially in the presence of high concentrations of toluene, xylene and petroleum hydrocarbons.

Depending on the measuring task and application, the available time and the required sensitivity, benzene can be directly measured on-site with portable GC, PID or FID, or sampled with active or passive charcoal or thermodesorption sampling systems for subsequent laboratory analysis. Sensitivities to benzene may vary by a factor of 1000 between PID, GC and FID ($\leqslant$100 ppb), and thermodesorption analysis (<0·5 ppb), however, either method provides constraints in terms of specificity (especially PID, FID), need for calibration, or time for getting the result.

Direct indicating colorimetric detector tubes provide an easy-to-use, cost-effective and reliable alternative to these methods. Some benzene detector tubes which feature detection limits at 0·2 ppm and lower limits of the measuring range at 0·5 ppm, however, show strong cross-sensitivities to other aromatics and/or aliphatics. Recent developments have now led to a detector tube (Dräger Tube, Benzene 0·5/c) displaying both high sensitivity (detection limit: 0·2 ppm) and high specificity for benzene. The two pre-layers of this tube trap more than 4000 ppm of cross-interfering substances, like toluene (100 ppm) and xylene (100 ppm), ethylbenzene (1000 ppm), petroleum hydrocarbons (*n*-octane, 1500 ppm), and various other aromatics and aliphatics. This tube is widely applied for monitoring workplace atmospheres and for confined space entry measurements in the petrochemical industry. It may also be used for soil–air measurements, providing a fast and reliable tool for underground storage tank leakage detection, and for the specific differentiation between gasoline and diesel in conjunction with petroleum hydrocarbon measurements.

Development work is underway to improve the readability of the sometimes weak discoloration of this tube by using optoelectronics.

### Strategy for urban monitoring

**Frank R. Price**

*Sheffield City Council, Sheffield*

Air quality management in Sheffield, as with many other local authorities, began with the smoke control programmes of the 1960s and 70s. Changes to the fundamentals of domestic heating not only began the rudiments of the control of air quality but also began the monitoring networks which have brought us the AUN.

For many years the monitoring of air quality was an end in itself, and the development of a state-of-the-art monitoring network was almost an ultimate objective. This thinking altered in the latter part of the 1980s when, once the work of smoke control was over and the benefits of that work had been recognized, it was realized that data for data's sake was an empty aim that had little or no relevance to the new emphases being placed on public information, the impact of air quality on health and the development and maintenance of air quality standards.

This presentation outlined the development of air quality monitoring in Sheffield, detailed the current status of the network, identified the resource implications of local monitoring and discussed the application and effectiveness of the tools which are available to managers of local air quality.

### The United Kingdom air quality monitoring networks

**Clare Downing**
*AEA Technology, Abingdon*

The National Environmental Technology Centre, AEA Technology, manages or quality assures most of the UK's air quality monitoring networks on behalf of the Department of the Environment. The networks monitor a comprehensive range of pollutants, including sulphur dioxide, particulate matter, nitrogen oxides, ozone, carbon monoxide, hydrocarbons, lead and other metals, acid deposition and air toxics. The networks cover both the rural and urban environment and optimize different measurement techniques to meet the diverse network needs. The measurement techniques include both complex real-time automatic measurements for high resolution data and cost-effective sampler based methods to provide long-term measurements over large areas of the UK.

The UK's air quality monitoring programmes have been designed to:

(1) Provide an understanding of air quality problems in order that cost-effective policies and solutions can be developed.
(2) Assess how far standards and targets are being achieved.
(3) Provide public information on current and forecast future air quality.
(4) Assist the assessment of personal exposure to air pollutants.

The presentation briefly described the structure and sampling methods used in each of the networks and discussed some examples of results and how the data are used to assess whether standards, or other targets, such as critical loads have been achieved.

### A new piezo-electric ozone monitor

**E. de Saeger and P. Pérez Ballesta**
*Ispra Environment Institute, Italy*

Diffusive samplers are a valid and cost-effective alternative to conventional techniques for ambient air measurements. They are particularly well adapted for long-term averaging measurements and their use will be recommended by the coming EU Directive on Air Quality Assessment and Management for various applications. The poor time resolution of existing diffusive samplers are a drawback when fast response times are required. This is particularly the case for ozone measurements for which air quality standards are based on hourly, four-hourly and eight-hourly measurements. A lower detection limit is then necessary and this can be achieved by adapting the design of the sampler or by increasing the sensitivity of the detection. A combination of the diffusive sampling principle with a piezo-electric sensor offers a promising future option.

The ozone sampler currently being developed by the authors' laboratory consists of a glass tube, at one end of which a specific ozone sensor is mounted. The sensor is constituted by a quartz oscillator, on the surface of which an ozone absorbing polymer film has been deposited. The polymer film reacts with the ozone molecule specifically, fixing one oxygen atom, and increases its mass. The variation in mass is detected by measuring the decrease of the quartz oscillation frequency, before and after the sampler exposure. This kind of piezo-electric sensor is also called Quartz Micro Balance (QMB). The high sensitivity of the QMB sensor is achieved by operating at high oscillating frequencies (10 MHz range). With a detection limit of a few picograms of ozone, the exposure time of the detector can be reduced to a few hours, allowing for the monitoring of ozone during photo-chemical episodes.

Piezo-electric sensors like the QMB seem to be well adapted for the development of fast response diffusive samplers for the measurement of ozone but also for other pollutants. In addition, this technique will also allow for the development of diffusive samplers with continuous reading of the measurements during sampling.

### Portable XRF systems for pollution monitoring

**Margaret West**
*Sheffield Hallam University, Sheffield*

X-Ray Fluorescence (XRF) spectrometry is an established technique for the analysis of samples associated with both workplace and environmental monitoring. However, in common with all sophisticated instrumentation, the spectrometer can only provide analytical data on samples submitted to the laboratory. The recent introduction of a field portable XRF analyser provides an opportunity to take the spectrometer to the sample. The performance of an energy dispersive portable system was compared with a conventional wavelength dispersive spectrometer with particular reference made to its application in the assessment of inorganic airborne and surface contamination.

### The electret dust sampler

**R. C. Brown**
*Health and Safety Executive, Sheffield*

Passive personal samplers for vapours do not require a pump, which makes them light in weight and very acceptable to wearers. A passive sampler for dust has the same advantage. Passive sampling requires motion of particles towards a collector surface. However, diffusive motion, which is a very effective transport process for molecules, is ineffective even for very fine dust particles. In the electret-based passive dust sampler the force causing capture is electrostatic attraction of the dust particles to the sampling surface, which is an electret. Electrets are polymers that carry a permanent electric charge, and certain polymers, for example polypropylene, can hold a charge that is stable to thermal decay for a period of years. With the use of such polymers a sampling region with a permanent electric field of approximately $10^5$ V/m is possible. The drift velocity of particles under the influence of an electric field and therefore the sampling rate is independent of the convective velocity of the air carrying the particles into and out of the sampling region. This is an important property that the electret sampler shares with passive vapour samplers. Both laboratory trials and field trials have been carried out on a prototype electret-based passive sampler. Field trials in which the electret-based sampler and a conventional inhalable dust sampler are worn by the same operative in metal-processing industries have shown good correlation between measurements made with the two devices. The sampling rate of the electret-based device depends upon the electrical mobility of the particles being sampled, and this means that a calibration factor is required in order for the device to give absolute measurements. This calibration factor can be obtained by separate measurements of electrical mobility or from field-trial measurements carried out with the device itself. The author has found that the latter has proved more satisfactory. Gravimetric measurements carried out in the pigment-processing industries suggest that the calibration factor is relatively constant with time, but that the scatter of results needs to be reduced. Preliminary measurements in which the device has been used for sampling asbestos fibres have shown good correlation with the results from conventional samplers.

### Portable monitors for asbestos

**Garry Burdett**
*Health and Safety Executive, Sheffield*

The use of portable monitors which give a real-time read out of the airborne fibre concentration has been pursued for many years as an attractive alternative to the manual counting of filtered samples by optical phase contrast microscopy (PCM). All the methods attempted have generally relied on being able to first align fibres and then use light scattering to detect the fibres by virtue of the scattering produced. Two instruments are currently marketed as portable fibre monitors.

The MIE FM7400 is the latest version of the FAM 1 which was initially developed at NIOSH in the USA before being further developed in industry as a commercial instrument. The FM-7400 is factory calibrated to agree with PCM analysis of membrane filter collected fibres, following the NIOSH 7400 method. The Fibrecheck FC2 monitor is a recently developed UK fibre monitor, which became available in 1995. Like the FM-7400, it claims to monitor asbestos and man-made mineral fibre (MMMFs) airborne concentrations.

In a series of recent experiments where fibrous aerosols were generated in a dust box it was decided to monitor the aerosol using the two monitors and to compare the results to the UK method for asbestos monitoring (MDHS 39/3) by membrane filter sampling and PCM analysis. Aerosols of long fibre crocidolite, UICC amosite, and UICC Chrysotile 'A' were generated using a fluidized bed aerosol generator and the results compared. In addition, the FC2 monitor has been used to monitor the airborne release of fibres from a variety of MMMFs in a larger dust chamber. This gave an ideal opportunity to assess the actual performance of the instruments against what are relatively pure fibre aerosols.

The results showed that there continue to be problems with fibre monitors, even under ideal test conditions. The monitors can only be used to give an indication of whether levels are increasing or decreasing and must be regularly calibrated against a side-by-side filter analysed by PCM. The use and performance of these instruments for field monitoring were discussed.

The following are brief summaries of posters which were presented at this meeting.

### Poster: Analysis of trace levels of VOCs in environmental samples by ATD-GC-MS

**M. R. Allen, A. Braithwaite and C. C. Hills**
*Nottingham Trent University, Nottingham*

The odours and perceived health risks associated with emissions from landfill sites often lead to complaints from local residents. This has led to an increased interest in monitoring ambient air surrounding such facilities. Odours result from the release of trace gaseous components such as halocarbons, organosulphurs, esters, alcohols, alkanes and aromatics into the atmosphere. In order to identify and quantify the low levels of VOCs present in landfill gas, a suitable pre-concentration technique and analytical method is required that is suitable for analytes with a wide boiling point range. A method for the determination using automated thermal desorption-gas chromatography-mass spectrometry has been developed. The method includes pre-concentration of the trace VOCs using sample adsorption tubes containing three adsorbents packed in series, Tenax TA, Chromosorb 102, and Carbosieve SIII. Breakthrough volumes and recovery measurements were carried out on landfill gas samples of up to 1 l. Breakthrough was not observed on any of the samples taken and recovery for all the components was found to be approximately 100%. Over 125 VOCs have been identified in landfill gas. The work that was reported in this poster is continuing to accumulate a database of information to establish trends and for inter-comparison of the emissions from a range of sites with varied tipping histories.

### Poster: Water and air quality monitoring using a novel electroluminescence sensor coupled to photo or sono energy sources

**M. J. Hepher, A. E. Burgess, J. Higgins, A. Savage and J. Baird**
*Glasgow Caledonian University, Glasgow*

A novel electroluminescence sensor and its application to water and air quality monitoring was described. The response of the sensor to selected pollutants within either the atmosphere or natural waterways can be used as an index of the water or air quality. The system uses chemical quenching of the electroluminescent luminol/hydrogen peroxide reaction. This is in marked contrast to the conventional chemical catalysed lumino/hydrogen peroxide reaction which utilise either cobalt ($Co^{2+}$) or horseradish peroxidise (HRPO) as the catalyst. The electroluminescence sensor consists of a fibre optic bundle (3 mm diameter) with cathode collar surrounding the fibre tip and an anode reflector plate, a few mm away from the fibre tip and parallel to it. Significant enhancement of the sensor signal can be obtained by coupling either UV radiation (200–350 nm) or sono energy (38 kHz) to the electroluminescence sensor. The operational characteristics, detection limits sensitivity and proposed improvements to the system were outlined.

### Poster: Environmental exposure to natural mineral fibres among inhabitants of houses in the vicinity of a serpentinite mine

**Edward Wiecek and Helena Woźniak**
*Nofer Institute of Occupational Medicine, Poland*

Serpentinite quarrying and processing is accompanied by remarkable emissions of mineral fibrous dust to the occupational and ambient air. The main aim of this paper was to determine concentration levels, fibre length distribution, and to assess health risk for the local residents. Three rural-type flats located 0·5–1·0 km away from the serpentinite mine and processing plant were selected for the study.

Indoor airborne total dust and fibre concentrations were determined by the gravimetric and phase-contrast optical microscopy methods, respectively. Fibre length distribution was determined with the MIE 7400 Fiber Monitor.

Airborne total dust concentration ranged from 65·8–387·5 $\mu g/m^3$ in winter and 70·0–145·5 $\mu g/m^3$ in summer. Mean yearly concentration of airborne dust was 155 $\mu g/m^3$. A concentration like this is approximately equal to the mean airborne dust concentration in a big industrial city. Fibre concentrations in the indoor air ranged from 2·3–10·00 fibres/l in winter and 4·8–27·0 fibres/l in summer. Mean yearly fibre concentration was estimated at 10·2 fibres/l. This was about 10 times more than the admissible fibre concentrations for the atmospheric air and corresponded to the fibrous particle concentrations in cities with heavy road traffic. The length of 55·9% fibres was less than 5 μm. The respirable fibres above 5 μm constituted 44·1% of total fibres.

It is extremely difficult to assess health risk level but, from experiments on animals, mortality data for urban areas with increased atmospheric air dust concentrations, and from the estimated cancer risk in urban areas exposed to fibrous (asbestos) dust, it may be inferred that life risk may be as high as 35 additional fatal cases per 100 000 inhabitants, including four additional deaths from lung cancer and mesothelioma.

### Poster: Exposure to toxic chemicals in the vicinity of municipal waste disposal

**Jan P. Gromiec and Barbara Romanowicz**
*Nofer Institute of Occupational Medicine, Poland*

Biodegradation of municipal wastes results in the emission of toxic gases and vapours and contamination of atmosphere in areas surrounding waste landfills. The purpose of the work presented in this poster was to identify toxic substances and to determine air contaminant concentrations in the vicinity of Lodz municipal waste disposal.

Twenty-four hour air samples were collected for six months at four different locations near the waste landfill for polar and nonpolar organic compounds. For comparison, air samples in Lodz residential areas were also collected. Compound identification was performed by a GC-MSD method, quantitative analysis involved GC-FID for nonpolar and GC-ECD for polar air pollutants. Low concentrations of aliphatic hydrocarbons, benzene, toluene, xylenes, chloroform, trichlorethane, trichloroethylene, tetrachloroethylene, carbon tetrachloride and chlorobenzene were identified in air samples. Mutagenic activity of atmospheric aerosols collected in the vicinity of the waste disposal was lower than that collected in residential areas.

**Poster: Fibre-optic based environmental exposure monitoring**

**T. E. Brook and R. Narayanaswamy**
*University of Manchester Institute of Science and Technology (UMIST), Manchester*

Heightened awareness of occupational exposure limits needing to be placed on various substances has led to a great increase in research to develop instruments for exposure monitoring. A growing area of research is that of fibre-optic based chemical sensors, to which chlorine gas monitoring has been applied. These sensors offer advantages over the conventional electrically based sensors in that they are resistant to electromagnetic interference, can be operated remotely in hazardous environments, are generally of rugged construction, require no electrical current at the sensing end, and are more resistant to chemical hazards.

A two-analyte system is currently under development for measuring both chlorine gas and relative humidity (the latter acts as an interferent to the chlorine sensor). The chlorine sensor is based on an silicone rubber encapsulated ruthenium (II) trisbipyridyl complex and utilizing fluorescence quenching as the measurement process. The RH sensor is based on a crystal violet–Nafion complex utilizing reflectance as the measurand. The poster presented results of the initial development of this system.

### The Automatic Methods Group meeting questionnaire

For the last four years those of you who have attended Automatic Methods Group (AMG) meetings will have been aware of the pressure to complete a two page questionnaire before leaving the hall. This is not a ploy to ensure that you miss a train, a means of determining your age (we known that it is 37 on average) or your qualifications but to let you tell us how well you liked the meeting and what you would like us to do in the future.

Despite 64–89% of you rating our meetings as 'Good' or better, we are frankly distressed that around 35% attendees have no formal association with The Royal Society of Chemistry (RSC), 50–60% are not in the RSC Analytical Division (AD) and between 83–94% are not members of the RSC AD Automatic Methods Group. At least join the AMG for the sake of your pocket!

If you are one of the unfortunate 43% who cannot attend meetings because of 'other commitments' please pass on its details to a friend. We now find that word of mouth is as effective an advertisement as our specialized direct mail shots.

The rationale for our meetings is greatly influenced by your answers. We now know that you prefer meetings on analytical applications rather than on analytical techniques. So, despite the AMG's name, there has been prime emphasis on the environment, and the monitoring of air and water. A torch for the automation of signal processing is being actively carried internationally by our Laboratory Information Management Systems subgroup. AMG meeting attendees most consistently claim an interest in sampling techniques as the basis for all subsequent analytical procedures. Yet the AMG committee retains the feeling that too few would attend such a general meeting. Maybe specific consultancy could meet your needs more effectively?

**J. R. P. Clarke**

### Forthcoming Meeting

**The academic–industrial interface: making the most of technology transfer**

**Royal Society of Chemistry Analytical Division: A Joint Meeting of the Western Region and the Automatic Methods Group, University of Bristol Tuesday 25 June 1996**

There is an increasing requirement for industrial relevance in academic research coupled with the outsourcing of more and more basic research by small companies and large corporations. This meeting will address the opportunities that have arisen out of this move towards industrial relevance and competitiveness, by highlighting existing collaborative arrangements, Government initiatives, and the benefits that can be gained by both industry and academe.

**Programme**

11.30 Registration

12.15 Lunch (provided)

13.30 Chairman's Introduction

13.40 From Corporate to Customer-focused: Changes in Industrial Analytical Technology Delivery
*Don White, BP Research, Sunbury*

14.05 Overview of Teaching Company Scheme
*Roland Burns, Plymouth Teaching Company Centre*

14.30 The Academic's View
*Professor Les Ebdon, University of Plymouth*

14.55 The Student's View
*Noel Brahma, Teaching Company Associate at PS Analytical*

15.20 Government Financial Support for Technology Transfer Activities
*Lyndon Davies, Lyndon Davies Associates*

15.45 Open Session *Chaired by Ken Leiper, Glaxo Pharmaceuticals*

16.30 End

**Registration details**

**The Academic–Industrial Interface: Making the Most of Technology Transfer**

**University of Bristol**

**Tuesday 25 June 1996**

Registration fee (please tick as appropriate)

| | | |
|---|---|---|
| Members of the Royal Society of Chemistry | £35 | ☐ |
| Non-members | £45 | ☐ |
| Students/Retired | £25 | ☐ |

Registration fee includes lunch.

To register for the above meeting please fill in this form and send, together with your registration fee, to:

Dr E H Evans
University of Plymouth
Department of Environmental Sciences
Drake Circus
Plymouth PL4 8AA

Tel: 01752 233040
Fax: 01752 233040

**Deadline 18 June 1996**

Make cheques payable to the Royal Society of Chemistry

---

I would like to register for the meeting entitled **The Academic–Industrial Interface: Making the Most of Technology Transfer**

Title: ____________ Name: ______________________________

Address: ______________________________

______________________________

______________________________

Tel.: ____________________ Fax: ____________________

Special dietary requirements: ______________________________

I enclose a remittance of £____________

Signed: ____________________ Date: ____________________

